# Veterinarians' attitudes, knowledge, and practices about antibiotic use in animals: questionnaire design and reliability

**DOI:** 10.3389/fvets.2025.1754010

**Published:** 2026-01-21

**Authors:** Ana Filipa Pereira, Leonardo de Rago, Jacinta Oliveira Pinho, Ana Isabel Plácido, Adolfo Figueiras, Fátima Roque, Maria Teresa Herdeiro, Ana Cláudia Coelho, Paula Alexandra Oliveira

**Affiliations:** 1 Department of Veterinary Sciences, School of Agrarian and Veterinary Sciences (ECAV), University of Trás-os-Montes and Alto Douro (UTAD), Vila Real, Portugal; 2 Veterinary Teaching Hospital, University of Trás-os-Montes and Alto Douro (HVUTAD), Vila Real, Portugal; 3 Department of Medical Sciences, Institute of Biomedicine (iBiMED), University of Aveiro, Campus Universitário de Santiago, Aveiro, Portugal; 4 BRIDGES - Biotechnology Research, Innovation and Design for Health Products, Polytechnic University of Guarda, Guarda, Portugal; 5 Department of Preventive Medicine and Public Health, Faculty of Pharmacy, University of Santiago de Compostela, Santiago de Compostela, Spain; 6 Consortium for Biomedical Research in Epidemiology and Public Health (CIBER en Epidemiología y Salud Pública-CIBERESP), Carlos III Health Institute, Madrid, Spain; 7 Health Research Institute of Santiago de Compostela (IDIS), Santiago de Compostela, Spain; 8 Associate Laboratory for Animal and Veterinary Sciences (AL4AnimalS), Faculty of Veterinary Medicine, University of Lisboa, Lisboa, Portugal; 9 Department of Veterinary Sciences, Animal and Veterinary Research Centre (CECAV), University of Trás-os-Montes and Alto Douro (UTAD), Vila Real, Portugal; 10 Centre for the Research and Technology of Agro-Environmental and Biological Sciences (CITAB), University of Trás-os-Montes and Alto Douro (UTAD), Vila Real, Portugal

**Keywords:** antibiotic stewardship, antimicrobial resistance, One Health, questionnaire design, questionnaire reliability, veterinarians

## Abstract

**Introduction:**

Antimicrobial resistance (AMR) is a global public health concern that requires a One Health approach. The role of veterinarians in promoting antimicrobial stewardship is essential for successful mitigation of AMR.

**Objective:**

This study aimed to design a self-administered questionnaire and evaluate its reliability as a tool to assess veterinarians' knowledge, perceptions, and attitudes regarding AMR and antibiotic prescription and use in animals.

**Methods:**

The questionnaire was developed based on a comprehensive review of relevant literature and by employing collective intelligence methodologies, including focus groups with veterinarians and pharmacists. For the pilot study, veterinarians working in the Northern region of Portugal were recruited. A test-retest was conducted with a 4-week interval. Reproducibility was determined with the intraclass correlation coefficient (ICC; 95% confidence interval) and internal consistency was calculated using Cronbach's alpha.

**Results:**

In total, 31 (out of 34) veterinarians completed the retest phase of the study. Four sections with scale-items were assessed for reliability, with ICC values ranging from 0.10 (*p* = 0.285) in Section 2 (AMR) to 0.85 (*p* < 0.001) in Section 4 (prescription and antibiotic use). The questionnaire achieved Cronbach's alpha coefficient values of 0.81 and 0.78 in test and retest, respectively. Based on ICC values and veterinarians' comments, some items were deleted or reformulated.

**Conclusion:**

The developed questionnaire is a reliable instrument capable of capturing veterinarians' knowledge, perceptions, and attitudes on AMR and antibiotic use.

## 1 Introduction

Worldwide, antimicrobial resistance (AMR) is recognized as a major public health threat, negatively impacting societies, healthcare systems, and economies, and hindering the effective treatment of infections (1). In humans, forecasts between 2022 and 2050 predict a global rise of 69.6% in deaths caused by AMR and of 67.0% AMR-associated deaths (2). Some major adverse consequences of AMR include poorer health outcomes, prolonged disability of surviving patients (3), increased risk of post-surgical procedure complications (3), and higher treatment expenses (4). In animals, welfare and therapeutic outcomes can also be impaired and, in food-producing animals, productivity may be reduced and economic gains may be compromised (5–7).

Drug-resistant pathogens can be transmitted at the human-animal interface and this is particularly impactful when the therapeutic effectiveness of critically important antibiotics is compromised (8). Although resistance to antibiotics is a phenomenon that occurs naturally, the selective proliferation of bacteria with inherent or acquired resistance is promoted when these drugs are used in the context of human and veterinary medicine, and agriculture practices (9–11), being aggravated by the inappropriate use of antibiotics (12). Evidence from European health agencies indicates that reduction in antibiotic consumption correlates with decreased rates of AMR (13). In 2021, Portugal consumption of antimicrobial medicines in humans and food-producing animals was of 101.8 and 149.9 mg/kg estimated biomass, respectively, compared to EU/EEA mean values of 125.0 and 92.6 mg/kg estimated biomass (13).

A consensus has emerged recognizing that effective control of AMR emergence and dissemination requires multisectoral collaborative actions that integrate expertise from human, animal, and environmental health sectors—the One Health approach (14–18). In the One Health context, veterinarians are key players in promoting good production practices (e.g., hygiene, biosecurity, and vaccination), ensuring responsible antibiotic prescription, and increasing awareness among farmers (19). Research demonstrates that the misuse of antibiotics is often associated with prescriber-related factors, including knowledge, practices, and attitudes, as well as extrinsic and farmer/owner-related factors (20, 21).

In order for the One Health strategy to have a global impact and effectively tackle AMR, it has first to be employed at the national and regional levels, creating a ripple effect (22). In Portugal, only a few studies addressing veterinarians' prescription practices of antibiotics have been conducted, with either companion animals, exotic species, or livestock being the focus species (23–25). Therefore, the aim of this study was to develop and assess the reliability of a self-administered questionnaire designed to capture and understand Portuguese veterinarians' behaviors, attitudes, and perceptions about AMR and antibiotic prescription and use. This will serve as a tool to collect data on the topic of AMR and antibiotic use from the veterinarians' perspective, allowing for the subsequent design and implementation of tailored educational interventions to promote antibiotic stewardship and contribute to global efforts against AMR.

## 2 Materials and methods

### 2.1 Ethics approval and consent to participate

The research protocol was reviewed and received ethics approval from the Ethics Committee of the University of Trás-os-Montes and Alto Douro (Ethical approval reference Doc23-CE-UTAD-2025, on 05/03/2025). Informed consent was obtained from all individual participants included in the study.

### 2.2 Questionnaire design

The questionnaire was developed in Portuguese language it was based on: (i) a comprehensive review of relevant literature (26–35); and (ii) collective intelligence methods, namely a focus group study exploring veterinarians' perceptions, attitudes, and knowledge about AMR and antibiotic use in food-producing animals, to maintain relevance to local practices, regulations, and cultural norms. Focus groups allow to refine previously known information and can also provide additional insights about a topic, rendering this method particularly useful for item development (36).

#### 2.2.1 Face and content validity

One psychology expert and one Portuguese language expert evaluated face validity parameters, namely grammar, syntax, organization, appropriateness, potential question bias, and logical sequence of the statements (37, 38).

To ensure clarity, relevance of items, and comprehensiveness, the questionnaire was reviewed by a panel of 12 experts in the areas of veterinary medicine, public health, pharmacy, pharmacology, and pharmacoepidemiology. Content validity defines to what extent the set of items fully represent all aspects to be measured (39, 40), allowing to assess the accuracy, terminology, completeness, and meaning of items.

### 2.3 Pilot study: test-retest

The study was conducted in the Northern Region of Portugal, from February to April 2025, and the target populations were veterinarians. No restrictions were imposed on practice type, age, gender, or years of professional experience. At least 30 participants were recruited by convenience sampling (41, 42). The consent to participate in the pilot study was obtained from the individual veterinarians. To assess reproducibility, a test-retest was conducted, where questionnaires were delivered to the same participating veterinarians at an interval of 4 weeks (43–45).

### 2.4 Psychometric tests and statistical analysis

Data was randomly checked to evaluate any potential transcription errors. Data was analyzed using IBM SPSS statistics for windows, version 30.0 (IBM Corp., New York, United States) and missing values were excluded from analysis. Descriptive statistics, including frequencies, percentages, means, and standard deviations, were applied to summarize the data.

In Sections 2, 3, 4, and 6, the reproducibility of this self-administered questionnaire was determined using the intraclass correlation coefficient (ICC), with the respective 95% confidence interval (CI) (46), computed by two-way analysis of variance (ANOVA), mixed-effects model and single measures (47, 48).

The internal consistency of the developed questionnaire was estimated through Cronbach's alpha coefficient, widely used as an objective measurement of internal consistency. This coefficient ranges from 0 to 1 and defines the extent to which items within a test or instrument consistently measure the same construct or concept. Values ≥ 0.70 were defined as satisfactory reliability for an instrument with ordinal outcome variables, such as Likert scales (49–51). The questionnaire was not a single construct and, thus, this coefficient was only calculated for the 46 items in Sections 2, 3 and 4.

A preliminary Kaiser-Meyer-Olkin (KMO) test was performed to determine sampling adequacy for exploratory factor analysis.

## 3 Results

### 3.1 Questionnaire design and face and content validity

**Figure 1:**
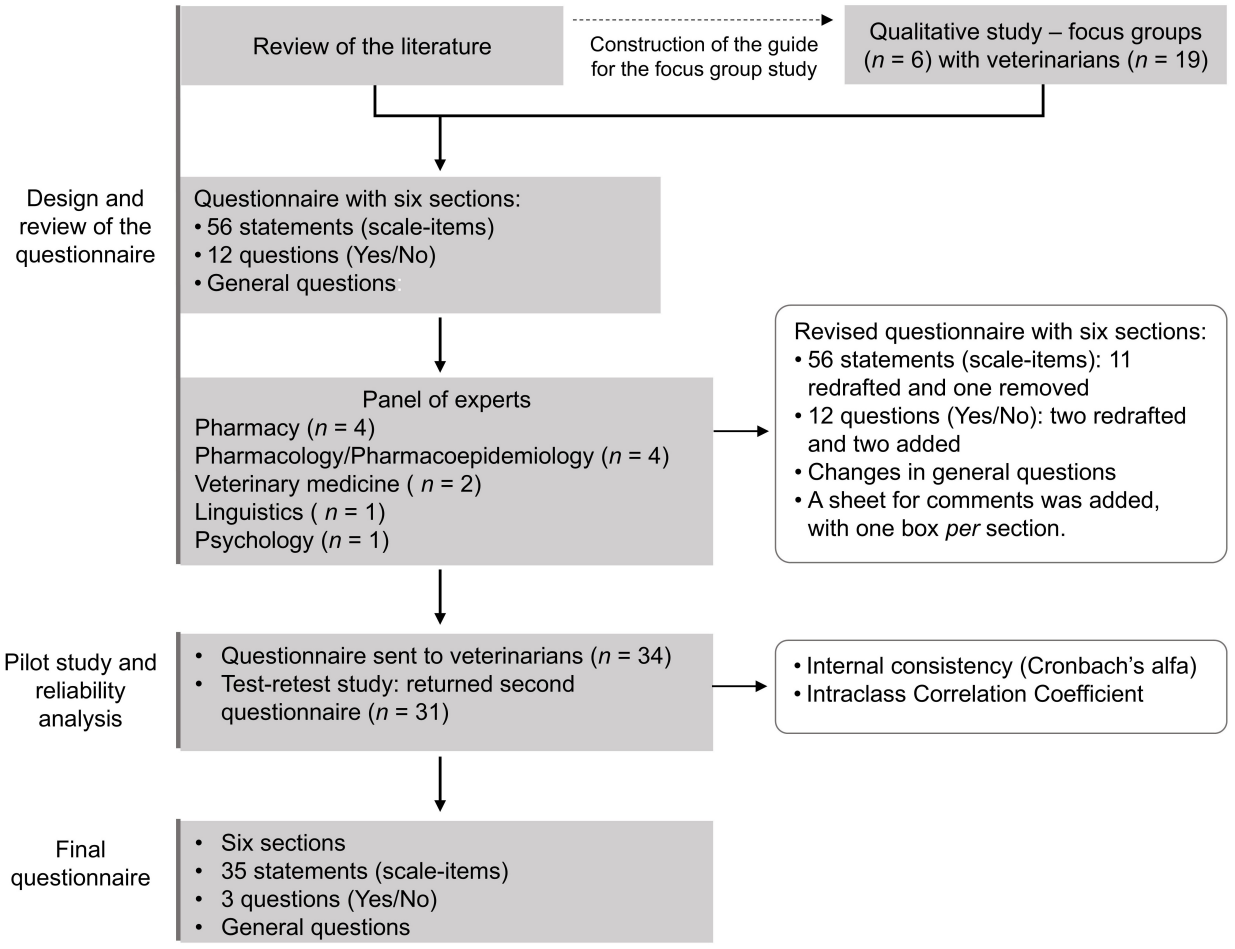
Study workflow.

In Figure 1 is shown the step-by-step process of questionnaire development.

A questionnaire was developed with six main sections (Supplementary material): (1) sociodemographic data: five questions on sex, age, type of practice, region of work, and years of experience; (2) AMR: two closed questions for quantitative evaluation of the knowledge about AMR and antibiotic use, with Yes/No answer or a five-point Likert scale (totally disagree to totally agree); (3) responsible use of antibiotics and disease prevention: seven closed questions of Yes/No, one closed question classified with five parameters from always to never, one closed question classified with a five-point Likert scale (totally disagree to totally agree), and one closed question classified in a scale from 0 – “must be eliminated from practice” to 10 – “must be always performed”; (4) prescription and use of antibiotics: one closed question of Yes/No, one closed question classified with five parameters from totally disagree to totally agree, two closed questions classified in a scale from 0 —“not important” to 10—“extremely important,” and one multiple choice question; (5) storage and disposal of antimicrobials: two Yes/No questions; and (6) training, communication, and information: one closed question classified with five parameters from always to never, one closed question classified with five parameters from totally disagree to totally agree, and one Yes/No question.

After revision by the panel of experts, 11 statements were reformulated and one removed since it was repeating information relayed by another item in the same section. In Section 5, the two Yes/No questions were redrafted as multiple option, single choice questions to account for differences that exist in storage and discarding of antibiotics depending on veterinarian's practice and farm setting. In this same section, two Yes/No questions were added to gain further insight into the topic of appropriate discarding of antibiotics and prescription practices of surplus drugs. The questionnaire was in paper format and, thus, a comment sheet was added as the final page, allowing veterinarians to write in the respective section's box.

### 3.2 Pilot study: internal consistency and reproducibility

From the 34 veterinarians invited for the test-retest study, 91.2% (31/34) completed the study. The sociodemographic characteristics of the retest participants are depicted in Table 1.

**Table 1:** Sociodemographic characteristics of participating veterinarians (*n* = 31).

**Characteristics**	***n* (%)**
**Sex**	
Male	10 (32.3)
Female	21 (67.7)
Age	36 (31.48)^*^
**Practice**	
Companion animals	20 (64.5)
Livestock (bovine, swine, small ruminants, or equine)	6 (19.4)
Exotic and/or wild animals	4 (12.9)
Mixed practice	1 (3.2)
**Region of work**	
North	28 (90.3)
Lisbon and Vale do Tejo	2 (6.5)
Alentejo	1 (3.2)
**Years of experience**	
>10 years	16 (51.6)
6–10 years	7 (22.6)
3–5 years	4 (12.9)
<3 years	4 (12.9)

^*^median (PCT25, PCT75).

For Sections 2, 3, 4, and 6, ICC values were calculated (Tables 2, 3). In Section 2, ICC values ranged from 0.10 (*p* = 0.285) for item (a) “Authorities overestimate the risk of antimicrobial resistance (AMR)” to 0.75 (*p* < 0.001) for item (d) “The ineffectiveness of antibiotic treatment is frequently observed in daily practice.” In Section 3, related to responsible use of antibiotics and disease prevention, the correlation coefficients ranged from 0.25 (*p* = 0.086) for item (g) “Adequate infrastructure” to 0.81 (*p* < 0.001) for item (a) “It is very useful in my daily practice.” In Section 4, focused on prescription and use of antibiotics, ICC values ranged from 0.29 (*p* = 0.056) for item (k) “Sometimes I use antibiotics even when diagnosis is not clear, i.e., the etiological agent is unknown” to 0.85 (*p* < 0.001) for item (xix) “Client/farmer/owner financial resources.” In Section 6, relating to training, communication, and information, obtained ICC values ranged from 0.31 for items (a) “Investment in digital platforms should be a priority (e.g.,: for information dissemination, guidelines consultation, prescription, among others)” (*p* = 0.043) and (c) “More awareness campaigns for AMR should be promoted for farmers/owners” (*p* = 0.044) to 0.44 (*p* = 0.006) for item (d) “Veterinarians have a responsibility to inform clients/farmers/owners about issues associated with AMR.”

**Table 2:** Intraclass correlation coefficient (ICC), with respective *p-value*, determined for each scale-item of Sections 2 and 3.

**Sections and items**	**ICC (95% CI)**	***p-value***
**Section 2. Antimicrobial resistance (AMR)** *Please indicate your level of agreement with each of the following statements, on a scale from 0 to 10 (0 – totally disagree; 10 – totally agree)*		
(a) Authorities overestimate the risk of antimicrobial resistance (AMR)^*^	0.10 (−0.25 to 0.44)	0.285
(b) Veterinarians play an essential role in protecting public health	0.42 (0.08–0.67)	0.009
(c) The potential contribution of antibiotic use in veterinary medicine for the development of resistance in humans is concerning^#^	0.58 (0.29–0.77)	<0.001
(d) The ineffectiveness of antibiotic treatment is frequently observed in daily practice	0.75 (0.54–0.87)	<0.001
(e) The current use of antibiotics in animals is too high	0.73 (0.52–0.86)	<0.001
(f) The access to antibiotics without a prescription is concerning^#^	0.34 (−0.01 to 0.62)	0.028
(g) The use of antibiotics in veterinary medicine has been decreasing over the years	0.65 (0.38–0.81)	<0.001
(h) The pharmaceutical industry has responsibility in the high use of antibiotics in veterinary medicine.	0.69 (0.44–0.84)	<0.001
(i) There should be greater oversight/monitoring by the competent authorities.	0.67 (0.42–0.83)	<0.001
**Section 3. Responsible use of antibiotics and disease prevention** *Please indicate your level of agreement with the following statements relating to the General Directorate for Food and Veterinary's (DGAV) online prescription platform, the Portuguese Electronic Veterinary Prescription Platform (PEMV) (totally disagree, disagree, neutral, agree, totally agree)*		
(a) It is very useful in my daily practice	0.81 (0.61–0.92)	<0.001
(b) It is intuitive and easy to use	0.79 (0.57–0.91)	<0.001
(c) PEMV is a hindrance in my daily practice^#^	0.59 (0.24–0.80)	0.001
(d) PEMV must be improved	0.75 (0.50–0.89)	<0.001
(e) PEMV should be available as a mobile app^*^	0.53 (0.15–0.77)	0.004
*Based on your knowledge and experience, on a scale of 0 to 10, please rate the following strategies and interventions aimed at preventing or reducing the occurrence of diseases in animals (0 – should be eliminated from practice; 5 – indifferent to implement; 10 – practice that should always be performed)^#^*		
(f) Vaccination	0.70 (0.46–0.84)	<0.001
(g) Adequate infrastructure	0.25 (0.11–0.55)	0.086
(h) Adequate nutrition	0.46 (0.13–0.70)	0.004
(i) Adequate hygiene conditions	0.54 (0.23–0.75)	<0.001
(j) Implementation of biosecurity protocols	0.56 (0.28–0.77)	<0.001
(k) Training sessions for the client/farmer	0.76 (0.56–0.88)	<0.001

^*^This item was removed. ^#^This item was reformulated.

**Table 3:** Intraclass correlation coefficient (ICC), with respective *p-value*, determined for each scale-item of Sections 4 and 6.

**Sections and items**	**ICC (95% CI)**	***p*-*value***
**Section 4. Prescription and use of antibiotics** *Please indicate your level of agreement with each of the following statements, on a scale of 0 to 10 (0 – totally disagree; 10 – totally agree)*		
(a) The correct use of antibiotics in livestock is important to mitigate AMR^*^	0.46 (0.13–0.70)	0.004
(b) The correct use of antibiotics in companion animals is important to mitigate AMR^*^	0.55 (0.25–0.75)	<0.001
(c) I try to reduce the use of antibiotics as much as possible^*^	0.73 (0.51–0.86)	<0.001
(d) During my practice, I often consider other therapeutic options before using antibiotics^*^	0.50 (0.19–0.72)	0.002
(e) I have sufficient resources and information for the correct use of antibiotics^#^	0.55 (0.25–0.75)	<0.001
(f) I am in favor of using antibiotics for prophylaxis^*^	0.36 (0.01–0.63)	0.021
(g) I am in favor of using antibiotics for metaphylaxis^*^	0.76 (0.56–0.88)	<0.001
(h) When antibiotic treatment fails, I tend to increase the dose and/or the duration of treatment	0.80 (0.63–0.90)	<0.001
(i) When antibiotic treatment fails, I tend to change the class of antibiotic	0.59 (0.30–0.78)	<0.001
(j) I always collect samples for susceptibility testing to choose the most appropriate antibiotic	0.81 (0.64–0.90)	<0.001
(k) Sometimes I use antibiotics even when diagnosis is not clear, i.e., the etiological agent is unknown^#^	0.29 (−0.07 to 0.58)	0.056
(l) I consider it an obligation to inform the client/farmer/owner about the proper use of antibiotics (e.g., dose, withdrawal period, route of administration)^#^	0.59 (0.31–0.78)	<0.001
**4.2.1 Factors related to the antibiotic** ^**#**^		
(i) Availability of the antibiotic	0.64 (0.37–0.81)	<0.001
(ii) Cost of the antibiotic	0.75 (0.55–0.87)	<0.001
(iii) Withdrawal period	0.71 (0.47–0.85)	<0.001
(iv) Spectrum of activity	0.65 (0.39–0.82)	<0.001
(v) Administration route	0.57 (0.27–0.77)	<0.001
(vi) Number of administrations	0.56 (0.26–0.76)	<0.001
(vii) Information on the summary of product characteristics	0.73 (0.52–0.86)	<0.001
(viii) Previous experience with the same active substance	0.41 (0.09–0.67)	0.008
(ix) Testimonials from veterinary colleagues	0.71 (0.48–0.85)	<0.001
(x) Advertisement/Catalogs	0.58 (0.28–0.77)	<0.001
(xi) Peer-reviewed scientific literature	0.61 (0.33–0.79)	<0.001
(xii) Associated side effects	0.37 (0.03–0.64)	0.017
**4.2.2 Factors related to the farm and/or the client/farmer/owner** ^**#**^		
(xiii) Husbandry conditions and adequate biosecurity measures	0.53 (0.21–0.75)	0.001
(xiv) Inexistence of vaccination	0.68 (0.43–0.84)	<0.001
(xv) Disease patterns observed on the farm	0.66 (0.38–0.83)	<0.001
(xvi) History of response to treatments	0.54 (0.22–0.75)	<0.001
(xvii) Client/farmer/owner goals	0.55 (0.24–0.76)	<0.001
(xviii) Client/farmer/owner preferences	0.74 (0.51–0.87)	<0.001
(xix) Client/farmer/owner financial resources	0.85 (0.70–0.93)	<0.001
**Section 6: Training, communication, and information** *Please indicate your level of agreement with the following statements (totally disagree, disagree, neutral, agree, totally agree)*		
(a) Investment in digital platforms should be a priority (e.g.,: for information dissemination, guidelines consultation, prescription…)^*^	0.31 (−0.05 to 0.59)	0.043
(b) Continuous training for veterinary professionals should be a priority^#^	0.38 (0.03–0.64)	0.017
(c) More awareness campaigns for AMR should be promoted for farmers/owners^#^	0.31 (−0.05–0.60)	0.044
(d) Veterinarians have a responsibility to inform clients/farmers/owners about issues associated with AMR^*^	0.44 (0.10–0.68)	0.006

^*^This item was removed. ^#^This item was reformulated.

Statements 2a, 4f, and 6a were removed due to ICC values of 0.10, 0.36, and 0.31, respectively. Although displaying moderate (3e and 4a, b, d), good (4c) and excellent (4g) ICC, these items were removed from the questionnaire following comments from the participants and due to redundancy.

The questionnaire achieved Cronbach's alpha coefficient values of 0.81 and 0.78 in test and retest, respectively.

The determined KMO was 0.41 and, since values < 0.5 are not acceptable for factor analysis (40, 52), we did not proceed with the exploratory factor analysis.

### 3.3 Comments and suggestions from the veterinarians

In total, 10 veterinarians provided comments and suggestions on the sheet designated for this purpose. None of the respondents commented about the comprehensiveness of the questionnaire. One veterinarian referred to the length of the questionnaire as a factor that could detract participation. Participants suggested the addition of questions related to AMR knowledge, susceptibility tests, availability of medicines for optimal clinical practice, reasons for low adherence to educational initiatives, and training for and monitoring of appropriate antibiotic use and disposal.

## 4 Discussion

Veterinarians are often gatekeepers of antibiotic use in animals and, under the One Health approach, essential for promoting antimicrobial stewardship (53–55). In this study, a questionnaire for identifying veterinarians' knowledge, perceptions, attitudes, and practices related to AMR and antibiotic use in animals was designed, demonstrating to be reliable, and reproducible.

Questionnaire's items were developed from a comprehensive review of relevant literature and a focus group study with livestock veterinarians and were required to be refined in terms of wording and content (40). Importantly, the type of question, language, and order of items may lead to biased responses. Thus, to ensure face and content validity, consultation with experts with different and relevant backgrounds was performed. For the design of comprehensive survey instruments, deductive and inductive methods are used (56). These correspond, respectively, to literature support and inputs from experts and/or target population, and to collective qualitative information. While the deductive method helps construct the theoretical basis for the items/domains, the inductive approach collects qualitative information from field experts and their individual experiences via interviews, focus groups, or field observations (56). In quantitative investigation, using focus groups for item development is particularly advantageous since this methodology is effective in generating new data and providing rich discussions, allowing participants to share particular insights and contrasting viewpoints (36, 57).

In the developed questionnaire, to collect knowledge, Yes/No questions were used, while for attitudes and practices, Likert scales were chosen. Likert-type scales (58–60) are useful to collect attitudes, perceptions, and opinions and are frequently used in human (38, 61–64) and veterinary medicine (26, 29). The number of options should provide the respondents with the opportunity to express positive, negative, and neutral views. In turn, this helps reduce response bias and enhance data reliability (65, 66). Ratings from 0–10 were also used (23), with one question providing the number “5” as a middle point anchor. Compared to 1–10 rating scales, the ones that use a 0–10 or 0–5–10 scales have lower levels of item non-response (67).

Obtained results demonstrate that the questionnaire was reliable and reproducible. Good reliability enhances the credibility of research findings (39, 68), and this is also emphasized in validated surveys on AMR and antibiotic use (24, 37, 38, 47, 61, 64, 69). Computed ICC values can be interpreted following published guidelines: values superior or equal to 0.75 indicate excellent inter-rater agreement; values from 0.60 to 0.74 indicate good agreement; values from 0.40 to 0.59 indicate fair to moderate agreement; and values lower than 0.40 indicate poor agreement (70).

Companion-animal veterinarians represented more than 60% of the target population, which is in line with statistics regarding veterinary profession in Portugal. According to the latest data from the representative institution of Veterinary Doctors (71), *n* = 7,541 practicing veterinarians are registered in Portugal, with *n* = 1,998 being located in Northern Portugal and *n* = 4,001 and *n* = 656 working with companion animals and livestock, respectively (71). Although most participants from the first phase also returned the second phase (retest) questionnaire (31 out of 34), the high number of questions was noted by one veterinarian. An extensive questionnaire, although important to obtain comprehensive data, may result in participants rushing through the questions and providing uniform, identical responses (72, 73). After considering the veterinarians' comments, questions were either deleted or reformulated and, thus, the length of the questionnaire was reduced while maintaining the quality of information retrieved.

The internal consistency, defined as the extent to which items within a test or instrument consistently measure the same construct, is frequently calculated using Cronbach's alpha (49). A value of 0.70 or higher indicates a good association among items, while values below 0.50 reflect a poor association. In turn, a Cronbach's alpha higher than 0.90 may suggest redundancy rather than a suitable level of consistency (74). In this study, for test and retest, Cronbach alpha values of 0.81 and 0.78 were obtained, respectively, demonstrating the internal consistency of the measurement instrument. Other studies on the design of surveys to address the same subject, either targeting physicians (75) or farmers (76), have obtained Cronbach values between 0.32 and 0.82.

The adequate reliability observed in our study could stem from the data initially gathered from the focus group sessions with veterinarians, as well as from other factors, namely: (i) the objective nature of the questions; (ii) the interest and concerns about AMR and inappropriate antibiotic use shared among veterinarians; (iii) the assessment of the instrument by an expert panel comprising veterinarians and pharmacists; (iv) the use of 5-point Likert and 0–10 radio-button scales (77, 78); and (v) the 4-week interval between test and retest (38, 45).

A current global public health priority is to preserve the therapeutic effectiveness of antibiotics by promoting their prudent and responsible use and, under the One Health approach, human and veterinary medicine settings are obligatory participants (16–18). As mentioned, there is a lack of surveys in Portugal with veterinarians on the topic of AMR. The existing ones (23–25) underline veterinarians as key antibiotic stewards and as One Health agents. Instruments that allow us to understand antibiotic prescription practices, knowledge, and drivers are necessary to effectively design and implement educational interventions targeting veterinarians to further support their antibiotic use decisions, while ensuring animal health, welfare, and productivity (25). Also, obtained data could help policymakers tailor national guidelines and legislation for the prudent use of antimicrobials in animals, contributing to the global efforts to tackle AMR emergence and spread.

The primary limitations of this study are the small size and convenience-based nature of the pilot study sample, as well as the region-specific veterinarian population (Northern of Portugal). Despite the limited number of participants and geographical distribution, the primary objective of the pilot study was to evaluate the reliability of the developed questionnaire in the early stages of the research. For this goal, the method of convenience sampling of 30 or more participants is frequently employed (41, 42). Although, in the present study, construct validity was not determined, this will be fully addressed in subsequent research with a larger sample number, during a full-scale cross-sectional study that encompasses all veterinarians working in Portugal as potential participants.

To conclude, a questionnaire, directed toward veterinarians and under the topic of AMR and antibiotic use, was designed and showed adequate reliability. This self-administered questionnaire could be useful to capture veterinarians' knowledge, perceptions, and attitudes, holding significant potential for the design of future educational interventions targeting this professional group. These initiatives, focusing on appropriate antibiotic prescription and use, could contribute to global efforts to tackle AMR and to promote public health.

## Data Availability

The raw data supporting the conclusions of this article will be made available by the authors, without undue reservation.
