# Supplementary material for: Veterinarians' attitudes, knowledge, and practices about antibiotic use in animals: questionnaire design and reliability
Source: Front Vet Sci. 2026 Jan 21;12:1754010. doi: 10.3389/fvets.2025.1754010 (PMC12867803; doi:10.3389/fvets.2025.1754010)
Supplement: Supplementary file 1 [file Data_Sheet_1.docx]

***Supplementary Material***

# **Supplementary Data**

**QUESTIONÁRIO**

**Antibióticos – Conhecimentos, Atitudes, Perceções e Práticas em relação às resistências e à prescrição**

1. **Caracterização sociodemográfica e profissional**
   1. Idade (anos)_______
   2. Género:

Homem

Mulher

Prefiro não responder

- 1. Qual o ramo principal de prática veterinária em que exerce?

Animais de companhia

Animais exóticos e/ou selvagens

Pecuária (bovino)

Pecuária (suíno)

Pecuária (pequenos ruminantes)

Pecuária (equídeo)

Prática mista

- 1. Em que região se situa o seu trabalho principal?

Norte

Centro

Alentejo

Algarve

Lisboa e Vale do Tejo

Açores

Madeira

- 1. Anos de experiência profissional:

Menos de 3 anos

3 a 5 anos

6 a 10 anos

Mais de 10 anos

1. **Resistências aos antibióticos (RAM)**

Nesta secção, pretendemos saber o seu conhecimento sobre a RAM e a sua opinião sobre a utilização de antibióticos em veterinária no geral.

- 1. Na sua prática, a RAM é um problema ao qual está sensível?

Sim

Não

- 1. Por favor, numa escala de 0 a 10, indique o seu grau de concordância com cada uma das seguintes afirmações. *(0 – discordo totalmente; 10 – concordo totalmente)*

| 1. As autoridades sobrestimam o risco de resistência a antibióticos (RAM). |  |
| --- | --- |
| 1. Os veterinários têm um papel essencial na proteção da saúde pública. |  |
| 1. A possível contribuição do uso de antibióticos em veterinária para o desenvolvimento de resistências em humanos é preocupante. |  |
| 1. A ineficácia do tratamento com antibióticos é frequentemente observada na prática diária. |  |
| 1. O consumo atual de antibióticos em animais é demasiado elevado. |  |
| 1. O acesso a antibióticos sem prescrição é preocupante. |  |
| 1. A utilização de antibióticos em veterinária tem vindo a reduzir ao longo dos anos. |  |
| 1. A indústria farmacêutica tem responsabilidade na elevada utilização de antibióticos em veterinária. |  |
| 1. Deveria haver uma maior fiscalização/monitorização por parte das entidades competentes. |  |

1. **Utilização prudente de antibióticos e prevenção da doença**
   1. Tem conhecimento das orientações internacionais para a utilização prudente de antimicrobianos, publicadas pela Organização Mundial de Saúde Animal (OMSA), Organização das Nações Unidas para a Alimentação e a Agricultura (FAO) e/ou Organização Mundial de Saúde (OMS)?

Sim

Não

- 1. Tem conhecimento da lista de agentes antimicrobianos de importância veterinária da OMSA?

Sim

Não

- 1. Tem conhecimento da lista de agentes antimicrobianos de importância médica da OMS?

Sim

Não

- 1. Tem conhecimento da categorização das classes de antibióticos para uso veterinário (grupos A, B, C e D) da Agência Europeia do Medicamento (EMA)?

Sim

Não

- 1. Com que frequência prescreve cada classe de antibióticos na sua prática clínica?

|  | Sempre | Frequentemente | Às vezes | Raramente | Nunca |
| --- | --- | --- | --- | --- | --- |
| Classe A (ex.: aminopenicilinas, monobactamos, rifampicina, carbapenemes, glicopeptídeos, estreptograminas) |  |  |  |  |  |
| Classe B (ex.: cefalosporinas 3ª e 4ª geração, polimixinas, fluoroquinolonas) |  |  |  |  |  |
| Classe C (ex.: aminoglicosídeos, cefalosporinas 1ª e 2ª geração, macrólidos, lincosamidas, aminopenicilinas com inibidores das lactamases beta) |  |  |  |  |  |
| Classe D (aminopenicilinas sem inibidores das lactamases beta, tetraciclinas, penicilinas naturais de espetro de ação estreito, sulfonamidas, polipeptídeos cíclicos) |  |  |  |  |  |

- 1. Por favor, indique os quatro antibióticos que, durante a sua prática, prescreve mais frequentemente:

(1)___________________________

(2)___________________________

(3)___________________________

(4)___________________________

- 1. Tem conhecimento das orientações da Direção Geral de Alimentação e Veterinária (DGAV) para a utilização prudente de antimicrobianos em animais?

Sim

Não

- 1. Considera que a legislação atual em Portugal é adequada para promover o uso prudente de antimicrobianos em veterinária?

Sim

Não

- 1. Utiliza a plataforma de prescrição eletrónica da DGAV, a PEMV?

Sim

Não

Se respondeu “Sim” na pergunta anterior, pode responder a esta questão. Caso tenha respondido “Não”, por favor passe à questão 3.11.

- 1. Em baixo estão algumas afirmações acerca da plataforma de prescrição eletrónica da DGAV, a PEMV. Por favor, para cada uma, indique o seu grau de concordância:

|  | Discordo totalmente | Discordo | Não concordo, nem discordo | Concordo | Concordo totalmente |
| --- | --- | --- | --- | --- | --- |
| 1. É muito útil na minha prática diária. |  |  |  |  |  |
| 1. É intuitiva e fácil de usar. |  |  |  |  |  |
| 1. A PEMV representa um entrave na minha prática diária. |  |  |  |  |  |
| 1. A PEMV deve ser melhorada. |  |  |  |  |  |
| 1. A PEMV deveria existir na forma de aplicação para telemóvel. |  |  |  |  |  |

- 1. De acordo com os seus conhecimentos e experiência, numa escala de 0 a 10, classifique as seguintes estratégias e intervenções destinadas a prevenir ou reduzir o aparecimento de doenças nos animais. *(0 - deve ser eliminada; 5 - é indiferente implementar; 10 - prática que deve ser sempre realizada)*

| Vacinação |  |
| --- | --- |
| Infraestruturas adequadas |  |
| Nutrição adequada |  |
| Condições de higiene adequadas |  |
| Implementação de protocolos de biossegurança |  |
| Ações de formação para o cliente/produtor |  |

1. **Prescrição/utilização de antibióticos em medicina veterinária**
   1. Quando prescreve um determinado antibiótico, tem em conta o risco de RAM?

Sim

Não

Por favor, numa escala de 0 a 10, indique o seu grau de concordância com cada uma das seguintes afirmações. *(0 – discordo totalmente; 10 – concordo totalmente)*

| 1. O uso correto de antibióticos em animais de produção é importante para mitigar a RAM. |  |
| --- | --- |
| 1. O uso correto de antibióticos em animais de companhia é importante para mitigar a RAM. |  |
| 1. Tento reduzir ao máximo a utilização de antibióticos. |  |
| 1. Durante a minha prática, costumo considerar outras opções terapêuticas antes de utilizar antibióticos. |  |
| 1. Tenho recursos e informação suficientes para uma utilização correta de antibióticos. |  |
| 1. Sou a favor do uso profilático de antibióticos. |  |
| 1. Sou a favor do uso metafilático de antibióticos. |  |
| 1. Quando o tratamento com antibiótico falha, tenho tendência a aumentar a dose e/ou o tempo de tratamento. |  |
| 1. Quando o tratamento com antibiótico falha, tenho tendência a mudar de classe de antibiótico. |  |
| 1. Recolho sempre amostra para testes de suscetibilidade para escolher o antibiótico mais adequado. |  |
| 1. Por vezes utilizo antibióticos mesmo quando o diagnóstico não é claro, ou seja, o agente etiológico é desconhecido. |  |
| 1. Considero ser uma obrigação informar o cliente/produtor/dono sobre a utilização adequada de antibióticos (ex.: dose, intervalo de segurança, via de administração). |  |

- 1. Nas tabelas em baixo estão alguns fatores que podem influenciar a decisão dos veterinários em prescrever antibióticos. Numa escala de 0 a 10, indique a importância de cada um na sua prática clínica. *(0 = nada importante, 5 = importante e 10 = muito importante)*
     1. **Fatores relacionados com o antibiótico**

| Disponibilidade do antibiótico |  |
| --- | --- |
| Custo do antibiótico |  |
| Intervalo de segurança |  |
| Espetro de atividade |  |
| Via de administração |  |
| Número de administrações |  |
| Indicações do resumo das características do medicamento veterinário (RCMV) |  |
| Experiência anterior com o mesmo princípio ativo |  |
| Testemunhos de colegas veterinários |  |
| Publicidade/Catálogos |  |
| Literatura científica reconhecida |  |
| Efeitos secundários associados |  |

- - 1. **Fatores relacionados com a exploração e/ou o cliente/produtor/dono**

| Condições estruturais e medidas de biossegurança adequadas |  |
| --- | --- |
| Inexistência de vacinação |  |
| Padrões de doença observados na exploração |  |
| Histórico de resposta aos tratamentos |  |
| Objetivos do cliente/produtor/dono |  |
| Preferências do cliente/produtor/dono |  |
| Recursos financeiros do cliente/produtor/dono |  |

- 1. Nos casos de falha terapêutica, quais pensa serem as causas mais prováveis? *(pode selecionar várias opções)*

O proprietário não seguiu as instruções do veterinário e/ou da guia de tratamento

Inexistência de condições adequadas de biossegurança

O agente patogénico é resistente ao antimicrobiano usado

Outro. Por favor, especifique__________________________________________________

___________________________________________________________________________

___________________________________________________________________________

1. **Acondicionamento e eliminação/descarte de antimicrobianos**
   1. Na sua prática, qual a percentagem dos seus clientes/produtores/donos que acondicionam, devidamente, os medicamentos veterinários prescritos?

Menos de 10%

11 a 30%

31 a 40%

41 a 60%

61 a 80%

Mais de 80%

Não aplicável

- 1. Na sua prática, qual a percentagem dos seus clientes/produtores/donos que eliminam, devidamente, os medicamentos veterinários sobrantes e/ou as seringas?

Menos de 10%

11 a 30%

31 a 40%

41 a 60%

61 a 80%

Mais de 80%

Não aplicável

- 1. Na sua prática, oferece o serviço de descarte e de recolha de medicamentos veterinários sobrantes e/ou seringas?

Sim

Não

Não aplicável

- 1. Na sua prática, faz a prescrição de medicamentos veterinários sobrantes?

Sim

Não

Não aplicável

1. **Formação, comunicação e informação**
   1. Por favor, indique com que frequência consulta/utiliza as seguintes fontes de informação relacionadas com a utilização/prescrição de antibióticos.

|  | Sempre | Frequentemente | Às vezes | Raramente | Nunca |
| --- | --- | --- | --- | --- | --- |
| Ações educacionais (ex.: seminários, workshops) |  |  |  |  |  |
| Literatura científica reconhecida |  |  |  |  |  |
| Empresas farmacêuticas |  |  |  |  |  |
| Internet |  |  |  |  |  |
| Outros colegas de veterinária |  |  |  |  |  |
| Testes laboratoriais (ex.: cultura, antibiograma) |  |  |  |  |  |
| Catálogos de produtos veterinários |  |  |  |  |  |
| Publicações do governo/DGAV |  |  |  |  |  |
| Experiência clínica |  |  |  |  |  |
| RCMV (Resumo das características do medicamento veterinário) |  |  |  |  |  |
| MedVet |  |  |  |  |  |
| Outras fontes |  |  |  |  |  |

- 1. Por favor, indique o seu grau de concordância com as seguintes afirmações:

|  | Discordo totalmente | Discordo | Não concordo, nem discordo | Concordo | Concordo totalmente |
| --- | --- | --- | --- | --- | --- |
| 1. O investimento em plataformas digitais deveria ser uma prioridade *(ex.: para divulgação de informação, consulta de guidelines, prescrição…)*. |  |  |  |  |  |
| 1. A formação contínua dos profissionais de veterinária deveria ser uma prioridade. |  |  |  |  |  |
| 1. Deveriam ser promovidas mais ações de sensibilização para a RAM entre os produtores/donos. |  |  |  |  |  |
| 1. Os veterinários têm a responsabilidade de informar os clientes/produtores/donos das questões associadas à RAM. |  |  |  |  |  |

- 1. Alguma vez recebeu formação sobre a RAM e a utilização prudente de antibióticos em veterinária?

Não, nunca

Sim, há menos de 1 ano

Sim, há menos de 3 anos

Sim, há menos de 5 anos

Sim, há menos de 10 anos

Sim, há 10 ou mais anos

- 1. Estaria disponível para participar em ações educacionais acerca da RAM e da utilização de antibióticos em veterinária?

Sim

Não

- 1. De forma a nos ser possível acompanhar, de forma anónima, o impacto deste estudo, pedimos-lhe que responda às duas perguntas seguintes:
     1. Mês e ano de nascimento da sua mãe________________________________________
     2. Indique o último algarismo do seu número de telemóvel_________________________

Muito obrigado pela sua participação
